# Variant cardiac transthyretin amyloidosis presenting as hypertrophic cardiomyopathy with left ventricular outflow tract obstruction: a case report

**DOI:** 10.1093/ehjcr/ytaf029

**Published:** 2025-01-23

**Authors:** Viktoria Höller, Viktoria Santner, Johannes Schmid, Andreas Zirlik, Nicolas Verheyen

**Affiliations:** Division of Cardiology, Department of Internal Medicine, Medical University of Graz, Auenbruggerplatz 15, 8036 Graz, Austria; Division of Cardiology, Department of Internal Medicine, Medical University of Graz, Auenbruggerplatz 15, 8036 Graz, Austria; Division of General Radiology, Department of Radiology, Medical University of Graz, Auenbruggerplatz 9, 8036 Graz, Austria; Division of Cardiology, Department of Internal Medicine, Medical University of Graz, Auenbruggerplatz 15, 8036 Graz, Austria; Division of Cardiology, Department of Internal Medicine, Medical University of Graz, Auenbruggerplatz 15, 8036 Graz, Austria

**Keywords:** Variant transthyretin amyloidosis, Left ventricular outflow tract obstruction, Hypertrophic cardiomyopathy, Differential diagnosis, Genetic testing, Case report

## Abstract

**Background:**

Left ventricular outflow tract obstruction (LVOTO) is common in hypertrophic cardiomyopathy (HCM) but has not been reported in hereditary transthyretin amyloidosis (ATTRv).

**Case summary:**

Here, we describe a 67-year-old male patient with a hypertrophic phenotype who was initially diagnosed with LVOTO attributed to HCM. Echocardiographic features included a hyperdynamic left ventricular ejection fraction, severe septal hypertrophy, and elongated residual mitral leaflets permitting their systolic anterior motion. The patient was childless, and family history was negative for any cardiovascular disease. Significant coronary artery disease was diagnosed. In cardiac magnetic resonance imaging, the concurrent presence of different late enhancement patterns initially prevented an unequivocal diagnosis. Due to progressive neuropathy, genetic testing for ATTRv was performed and identified a pathogenic variant in the *TTR* gene. Cardiac involvement was confirmed by amyloid scintigraphy with a Perugini score of 3, and treatment with ribonucleic acid silencing therapy was commenced.

**Discussion:**

The present case illustrates that LVOTO can occur in patients with ATTRv. While cardiac imaging pointed towards sarcomeric HCM, the coexisting red flags initiated re-evaluation of the diagnosis suggesting cardiac amyloidosis as a potential cause. The presence of non-cardiac red flags of transthyretin amyloidosis (ATTR) should initiate specific diagnostic work-up even if ATTR is unlikely based on cardiac imaging.

**Learning points**

- Hereditary cardiac transthyretin amyloidosis may present with asymmetrical left ventricular hypertrophy and left ventricular outflow tract obstruction.
- The presence of non-cardiac red flags of transthyretin amyloidosis should initiate further diagnostic work-up in the direction of amyloidosis, even if cardiac patterns are atypical.

## Introduction

In patients with cardiac hypertrophy, diagnosis of left ventricular outflow tract obstruction (LVOTO) is a key step in the diagnostic process and further management. Up to 70% of patients with sarcomeric hypertrophic cardiomyopathy (HCM) present with LVOTO, but it may also occur in phenocopies mimicking HCM.^1^ While LVOTO affects up to 4% of patients with light chain amyloidosis (AL),^2^ cardiac transthyretin amyloidosis (ATTR) is typically non-obstructive. The presence of LVOTO is therefore often considered a sign that excludes a diagnosis of ATTR amyloidosis. This case highlights that LVOTO should not automatically steer the diagnostic workup towards sarcomeric HCM.

## Summary figure

**Figure:**
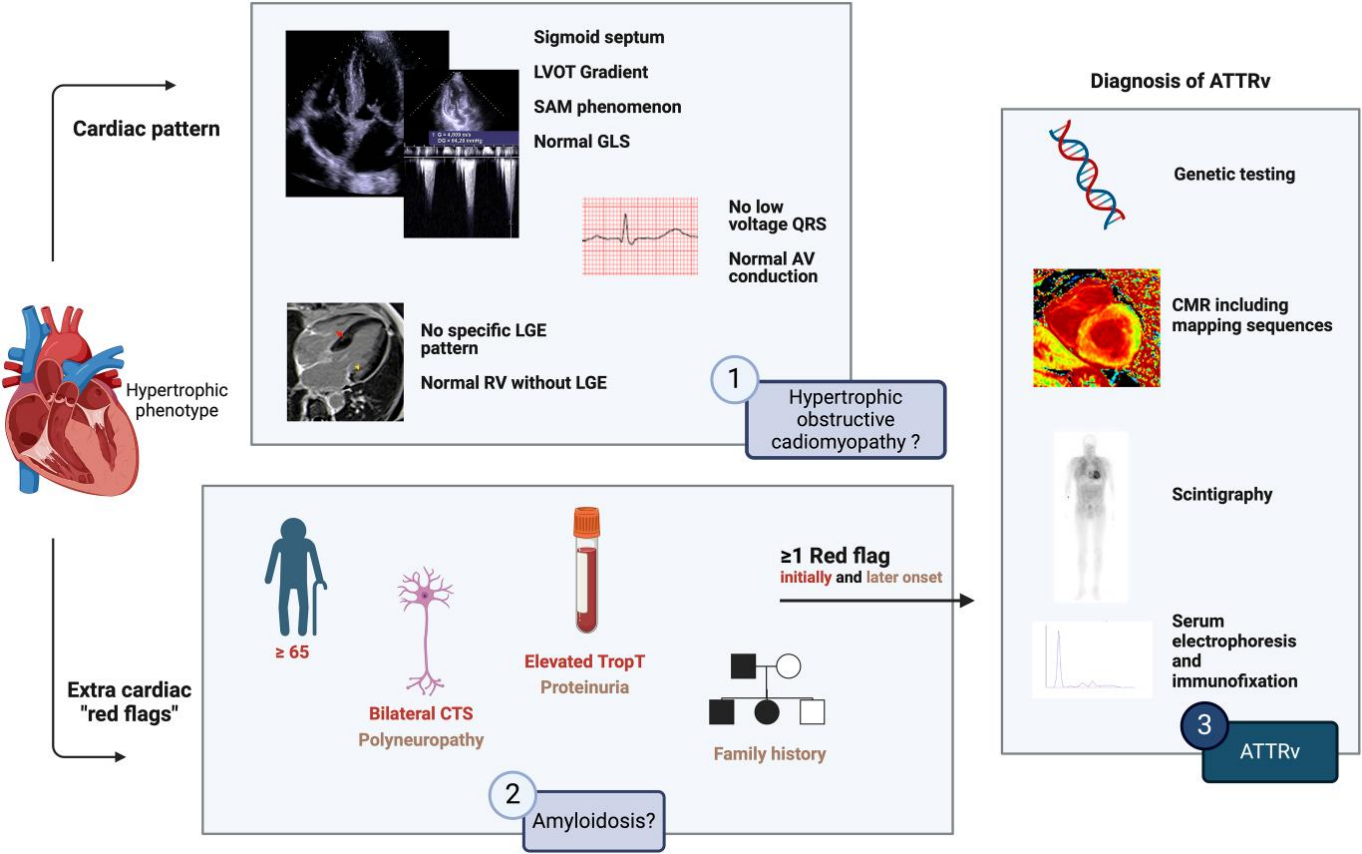


## Case presentation

In 2021, a 67-year-old male patient was referred for congestive heart failure in the presence of left ventricular hypertrophy (LVH). He presented with exertional dyspnoea [New York Heart Association (NYHA) Class II] and angina pectoris (Canadian Cardiovascular Society Class II) progressing during the last months. His medical history included operated bilateral carpal tunnel syndrome 15 years ago while symptoms of polyneuropathy were denied. Cardiovascular risk factors comprised arterial hypertension with an office blood pressure of 177/79 mmHg. Medication included bisoprolol 1.25 mg, amlodipine 5 mg, and candesartan 4 mg.

Levels of both N-terminal prohormone of brain natriuretic peptide and high sensitivity cardiac troponin T were elevated (119 pg/mL, reference range: <100 pg/mL; 28 pg/mL, reference range: <14 pg/mL), respectively, while serum creatinine was normal (0.93 mg/dL, reference range: <1 mg/dL). The 12-lead electrocardiogram (ECG) showed sinus bradycardia and left anterior hemiblock without evidence of low voltage or LVH (see Supplementary material online).

**Figure 1:**
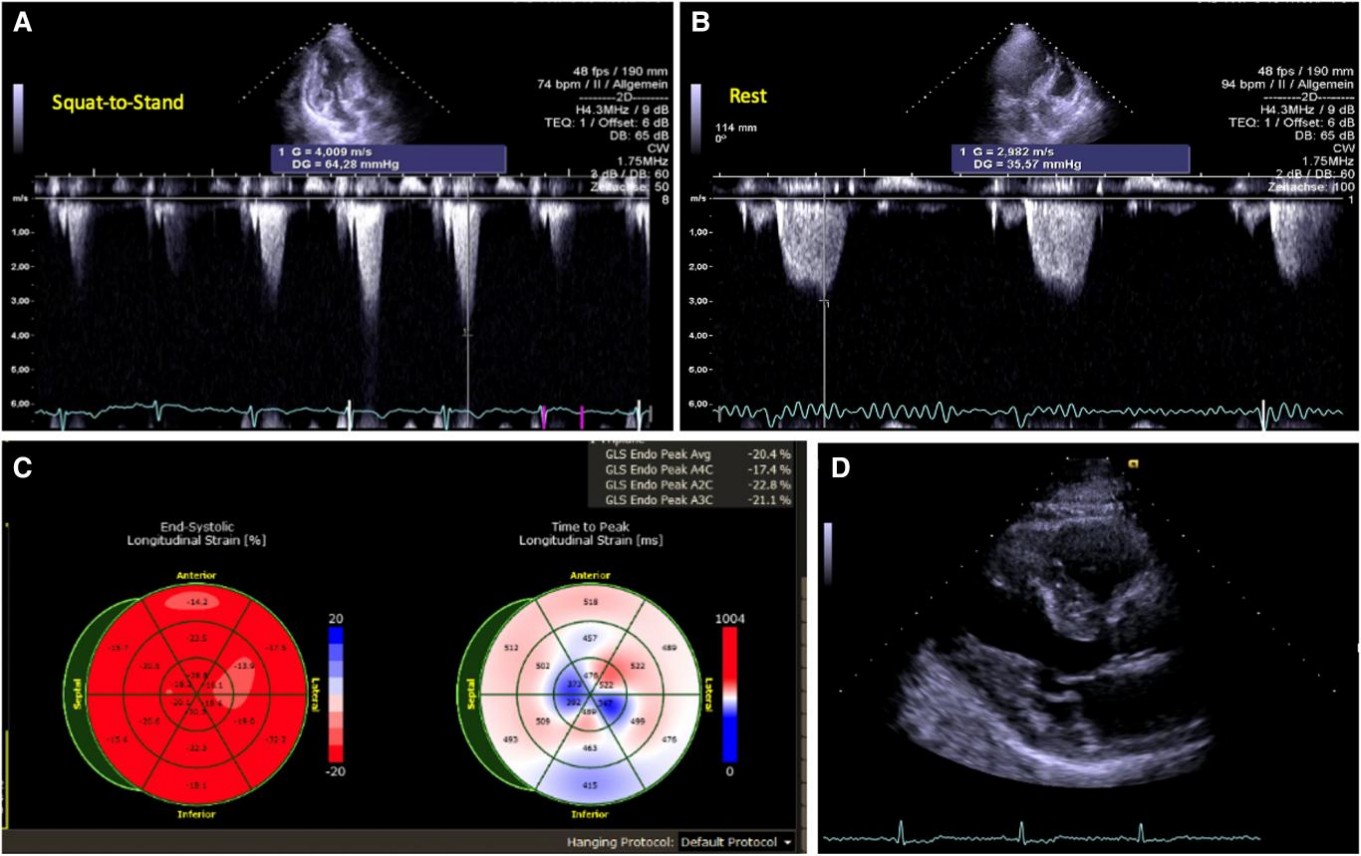
Echocardiography showed hypertrophy of the left ventricle with a maximum thickness of the left ventricular septum of 20 mm and of the posterior wall of 12 mm (*D*). Left ventricular ejection fraction was 77%, and myocardial deformation analysis did not indicate significant relative apical sparing or reduced global longitudinal strain in the predominant hypertrophic area (*C*). A resting gradient of 36 mmHg (*B*) with no change during Valsalva manoeuvre, and a gradient of 64 mmHg at squat-to-stand manoeuvre (*A*) was measured.

Transthoracic echocardiography revealed a maximal septal wall thickness of 20 mm with a sigmoid septal hypertrophy phenotype and a left ventricular ejection fraction of 77%. An incomplete systolic anterior motion (SAM) was observed at rest and during Valsalva manoeuvre, with a resting LVOT gradient of 36 mmHg. Forced provocation using the squat-to-stand manoeuvre induced complete SAM and significant LVOTO, with a peak gradient of 64 mmHg. Tip-to-septal distance was 5 mm indicating LVOTO, and the residual mitral leaflet was elongated to 14 mm. The free wall thickness of the right ventricle was 7.9 mm (*Figure 1*; Supplementary material online). In cardiac magnetic resonance (CMR) imaging, the concurrent presence of different disease patterns of late gadolinium enhancement (LGE) prevented an unequivocal CMR diagnosis. Additional mapping sequences were not performed initially.

No arrhythmia was detected in Holter ECG. Using the European Society of Cardiology HCM sudden cardiac death (SCD) risk calculator, an SCD risk of 1.89% was calculated yielding no indication for primary prophylactic implantable cardioverter defibrillator. Six-minute walking distance was 445 m. Initially, no genetic testing was performed, because the patient is childless, and family history was not indicative for hereditary cardiovascular disease. Results of diagnostic procedures were overall considered as compatible with the diagnosis of HCM complicated by LVOTO.

As recommended in the 2023 cardiomyopathy guidelines, bisoprolol was escalated.^3^ Coronary angiography revealed significant coronary artery disease. Functional flow reserve in the distal left anterior descending artery was 0.77, and implantation of a drug-eluting stents was performed. Due to critical stenosis in the mid-right coronary artery, another drug-eluting stent was implanted there. As the patient developed symptomatic bradycardia, the maximally tolerated bisoprolol dose remained at 1.25 mg daily. After the intervention, angina pectoris dissolved. However, 6-min walking distance did not improve (435 m), because of progressive pain and numbness in his legs.

In echocardiography, the provocable gradient was still present. Since the patient was asymptomatic at this point, amlodipine and candesartan were discontinued, due to their preload and afterload lowering effect, but pharmacological or septal reduction therapy options were not extended. Because of neurologically confirmed progredient polyneuropathy, additional testing for a mutation in the *TTR* gene was performed and showed a pathogenic variant (c.379A>Tp.(Ile127Phe)). In addition, 99mTc-DPD bone scintigraphy revealed significant myocardial radiotracer uptake equivalent to Perugini score 3.

**Figure 2:**
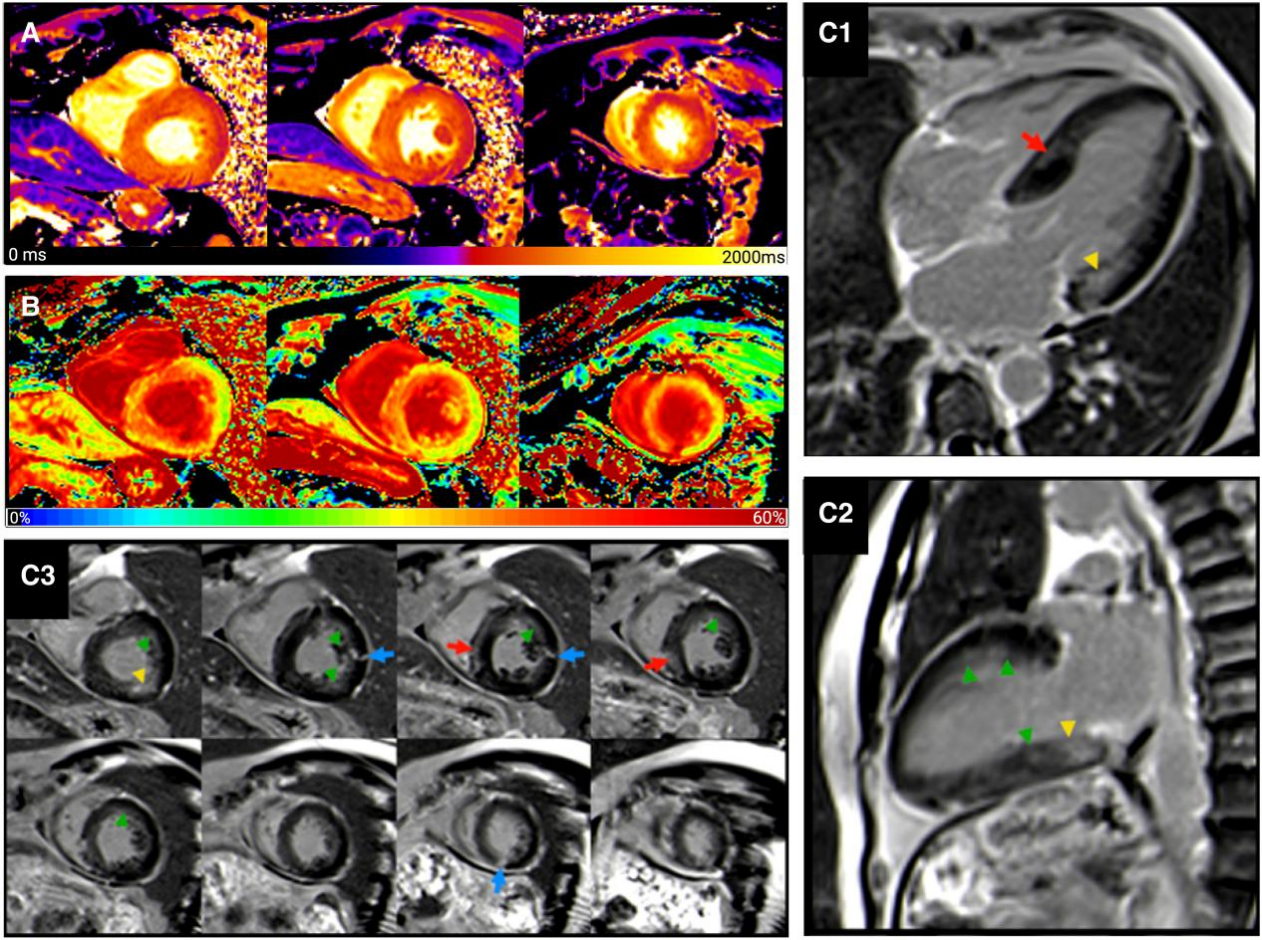
Cardiac magnetic resonance imaging. Native T1 maps (*A*) and extracellular volume maps (*B*) in a basal, mid-ventricular, and apical slice. Mean native myocardial T1 time per slice from base to apex was 1328, 1313, and 1285 ms (*z*-score 2.8, 2.4, and 1.6), and corresponding extracellular volume was 36, 34, and 35%. Late gadolinium enhancement (fast low-angle shot, phase-sensitive inversion-recovery) in a four-chamber view (*C*1), two-chamber view (*C*2), and short-axis stack from basal to apical (*C*3) showed indistinct subendocardial late gadolinium enhancement in the basal segments (green arrowheads) with hazy late gadolinium enhancement especially in the basal inferolateral segment (yellow arrowheads), patchy mid-myocardial septal late gadolinium enhancement (red arrows), and few small almost transmural foci of late gadolinium enhancement in the lateral and inferior wall (blue arrows). Additionally, the right ventricle presented normal in size and function without apparent wall thickening or detectable late gadolinium enhancement, while late gadolinium enhancement of the atrial walls could have been suspected in *C*1.

As assessed by free light chain assay, the kappa-to-lambda light chain ratio was normal (1.99, reference range: 1.35–2.65), and neither serum nor urine immunofixation electrophoresis could provide any evidence of present monoclonal paraproteins. The CMR was repeated, showing unchanged LGE with a complex combination of different LGE patterns, including (i) a basally predominant subendocardial to transmural hazy LGE, although in a distribution found in both ischaemic scar and amyloidosis, the hazy appearance is untypical for an ischaemic origin, (ii) a mid-wall septal LGE, compatible with non-ischaemic scar, and (iii) several focal spots of almost transmural LGE, possibly of ischaemic origin. Additional T1 and extracellular volume (ECV) maps revealed a baso-apical gradient of elevated native T1 and markedly elevated ECV, overall compatible with cardiac amyloidosis (*Figure 2*; Supplementary material online). Polyneuropathy Stage II due to ATTR with cardiac involvement was confirmed, and interdisciplinary disease management including a small interfering ribonucleic acid silencer therapy with vutrisiran was initiated.

**Figure 3:**
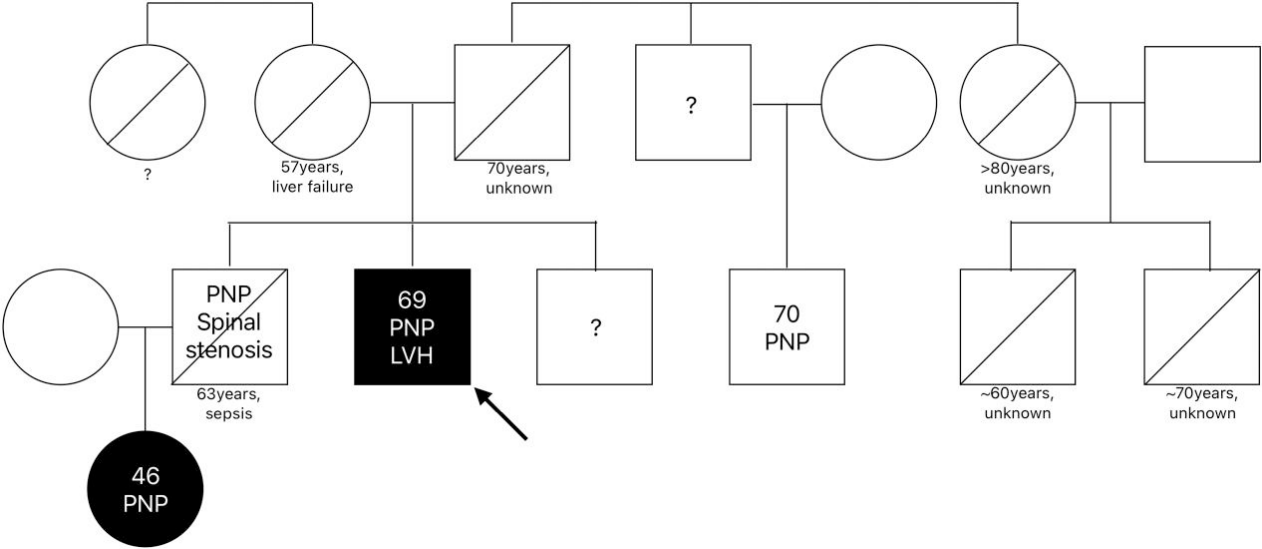
Family tree, obtained September 2023. The proband (arrow) was the index patient. Biological family relationships are denoted by squares (males) and circles (females), age, if known, is written inside. Positive genetic analysis for variant transthyretin amyloidosis is filled black. Death is shown by a crossed-out symbol, and age and cause of death are described below. Known cardiac or neurological symptoms are written into the squares and circles. LVH, left ventricular hypertrophy; PNP, polyneuropathy.

One year after therapy initiation, the patient presented in a cardiac asymptomatic condition, compared with NYHA Class II before admission, but clinically limited in his activities because of rapid progressive polyneuropathy. Shortly after the initiation of vutrisiran, there was a subjective improvement of polyneuropathy symptoms. In view of apparently normal filling pressures, beta-blocker therapy with 1.25 mg of bisoprolol was considered safe and was continued and well tolerated. No relevant LVOTO was present in echocardiographic follow-up, after adaptation of the medical therapy. A panel diagnostic for sarcomeric HCM was performed and did not reveal an additional mutation. Family cascade genetic was carried out (*Figure 3*) and led to confirmation of a positive genotype in a niece. He remains under regular annual follow-up in the cardiac outpatient clinic as well as in the neurological outpatient clinic.

## Discussion

Even if hereditary ATTR (ATTRv) is described in up to 4% of patients initially diagnosed with HCM,^4^ to our knowledge, this is the first report of a patient with confirmed ATTRv and LVOTO. Although LVOTO is classically associated with sarcomeric HCM, it can occur in various diseases associated with LVH if functional and structural prerequisites are fulfilled. These include high-normal or even hyperdynamic LV systolic function, severe LVH, and an elongated residual mitral leaflet past the coaptation point permitting the mitral valve to protrude into the LVOT, which was also present in the reported patient.^5^ Consequently, LVOTO has been reported as a complication in various diseases associated with severe LV hypertrophy and preserved ejection fraction including AL, Fabry disease, RASopathies, Tako-Tsubo cardiomyopathy, or aortic stenosis.^2,6–8^ Since cardiac amyloidosis is associated with impaired systolic function at the basal segments referred to as apical sparing, the presence of LVOTO in ATTR cardiomyopathy appears to a certain degree counterintuitive. In this case, cardiac imaging therefore rather pointed towards sarcomeric HCM, but the coexisting red flags initiated re-evaluation of the diagnosis suggesting cardiac amyloidosis as a potential cause. In fact, LVOTO has been reported as a rare complication in cardiac amyloidosis, both in ATTR and AL.^9,10^ Our study extends the current literature by reporting LVOTO in a patient with confirmed ATTRv.

Specific treatments for cardiac amyloidosis are only effective at improving cardiovascular outcomes at early stages, and therefore, early diagnosis is crucial.^11,12^ It remains unclear which medical treatment option is optimal if patients with cardiac amyloidosis develop symptomatic LVOTO. Even though alcohol septal ablation would be feasible,^13^ it is unknown whether patients with cardiac amyloidosis and LVOTO will benefit from septal reduction therapy^9^ and there is only one case report suggestive of haemodynamic improvement.^14^ In this indication, potential effects of specific medical treatment for obstructive HCM, for example with beta-blockers or cardiac myosin inhibitors, are also unknown, and the usage of beta-blocker therapy might worsen restrictive physiology in ATTR.^15^

In conclusion, the present case report points out that awareness of the potential coexistence of cardiac amyloidosis and LVOTO may permit more accurate diagnosis, particularly at early stages of amyloid disease, when more treatment options exist. While cardiac imaging initially suggested sarcomeric HCM as the underlying cause, the coexisting red flags initiated re-evaluation of the diagnosis indicating cardiac amyloidosis. The presence of non-cardiac red flags of ATTR amyloidosis should initiate specific diagnostic work-up even if ATTR is unlikely based on cardiac imaging.

## Supplementary Material

ytaf029_Supplementary_Data

## Data Availability

The data underlying this article are available in the article and in its online Supplementary material.

## Lead author biography

Dr Viktoria Höller studied medicine at the Medical University of Graz in Austria. She is currently a resident at the Division of Cardiology at the University Heart Centre in Graz specializing in heart failure. Her research focuses on hypertrophic cardiomyopathies, myocardial storage disease, and genetic cardiac disorders.

## Notes

**Consent:** The authors confirm that written consent for the submission and publication of this case report has been obtained from the patient in line with the COPE guidance.

**Funding:** None declared.
