# Supplementary material for: Variant cardiac transthyretin amyloidosis presenting as hypertrophic cardiomyopathy with left ventricular outflow tract obstruction: a case report
Source: Eur Heart J Case Rep. 2025 Jan 23;9(2):ytaf029. doi: 10.1093/ehjcr/ytaf029 (PMC11799945; doi:10.1093/ehjcr/ytaf029)
Supplement: ytaf029_Supplementary_Data [file ytaf029_supplementary_data.zip › Supplementary material captions_R1.docx]

**Supplementary material captions:**

**Supplementary Figure 1.** 12 channel electrocardiogram (ECG) with a paper speed of 50mm/s showing sinus bradycardia (59 bpm), left anterior hemiblock, normal atrioventricular conduction (PQ 184ms), QRS width of 106ms, persistent S waves and concordant terminal T waves.

**Supplementary Figure 2.** Cardiac magnet resonance imaging (CMR). Native T1 maps (A) and extracellular volume (ECV) maps (B) presented in Bullseye plots.

**Supplementary video 1.** 5-chamber-view in transthoracic echocardiography revealing left ventricular hypertrophy (maximum septal end-diastolic diameter of 20mm) with incomplete systolic anterior motion (SAM). Tip-to-septal distance was 5mm indicating LVOTO, and the residual mitral leaflet was elongated to 14mm.

**Supplementary video 2**: Cardiac magnet resonance imaging (CMR). Cine 4-chamber-view showing left ventricular hypertrophy predominantly at the basal septum (maximum end-diastolic myocardial thickness 20 mm), an enlarged left atrium, a regurgitant jet at the mitral valve and a mild SAM of the anterior mitral leaflet.
