# Supplementary figures and images for: Variant cardiac transthyretin amyloidosis presenting as hypertrophic cardiomyopathy with left ventricular outflow tract obstruction: a case report
Source: Eur Heart J Case Rep. 2025 Jan 23;9(2):ytaf029. doi: 10.1093/ehjcr/ytaf029 (PMC11799945; doi:10.1093/ehjcr/ytaf029)

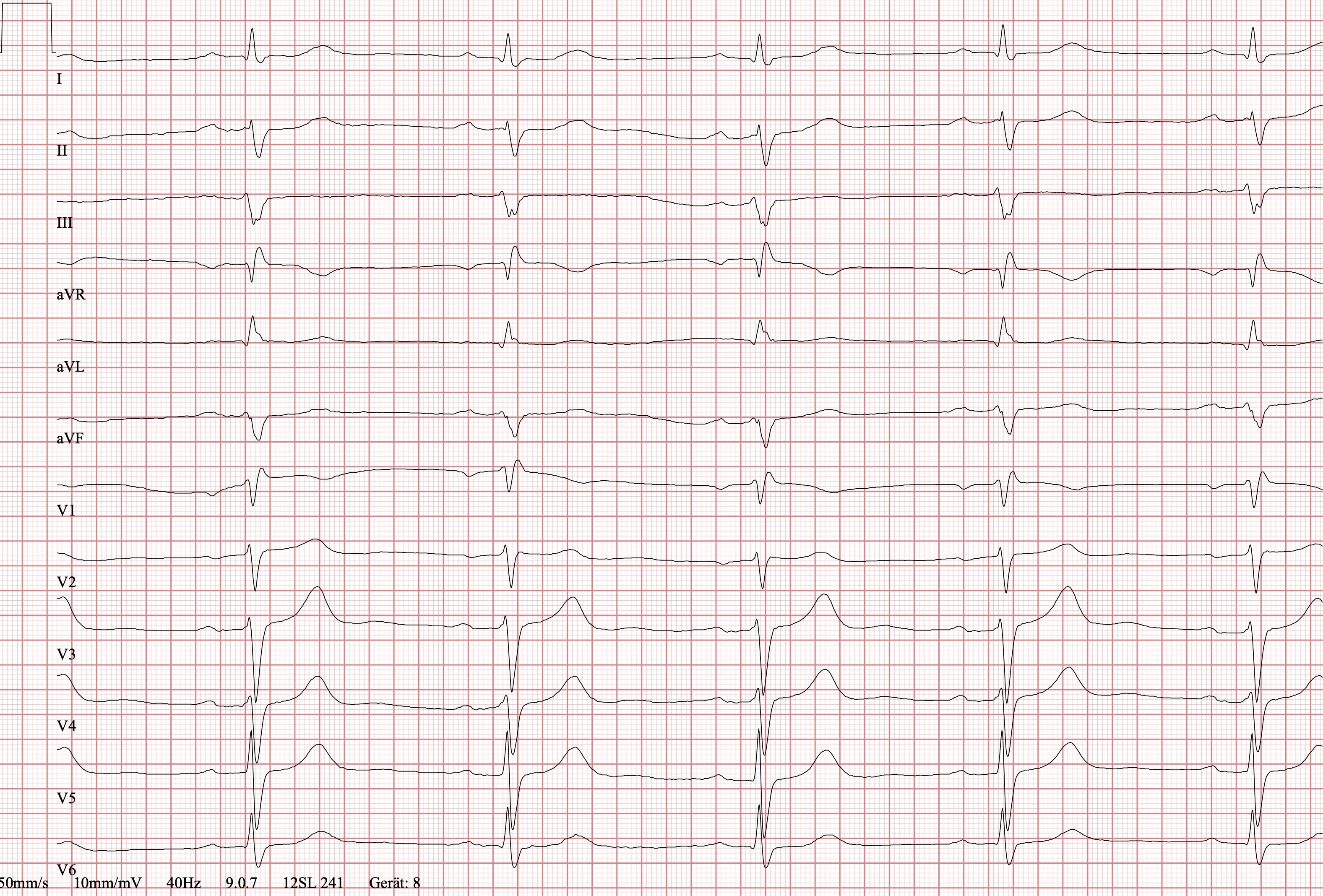

Supplement: ytaf029_Supplementary_Data [file ytaf029_supplementary_data.zip › Supplementary figure 1_R2.jpg]

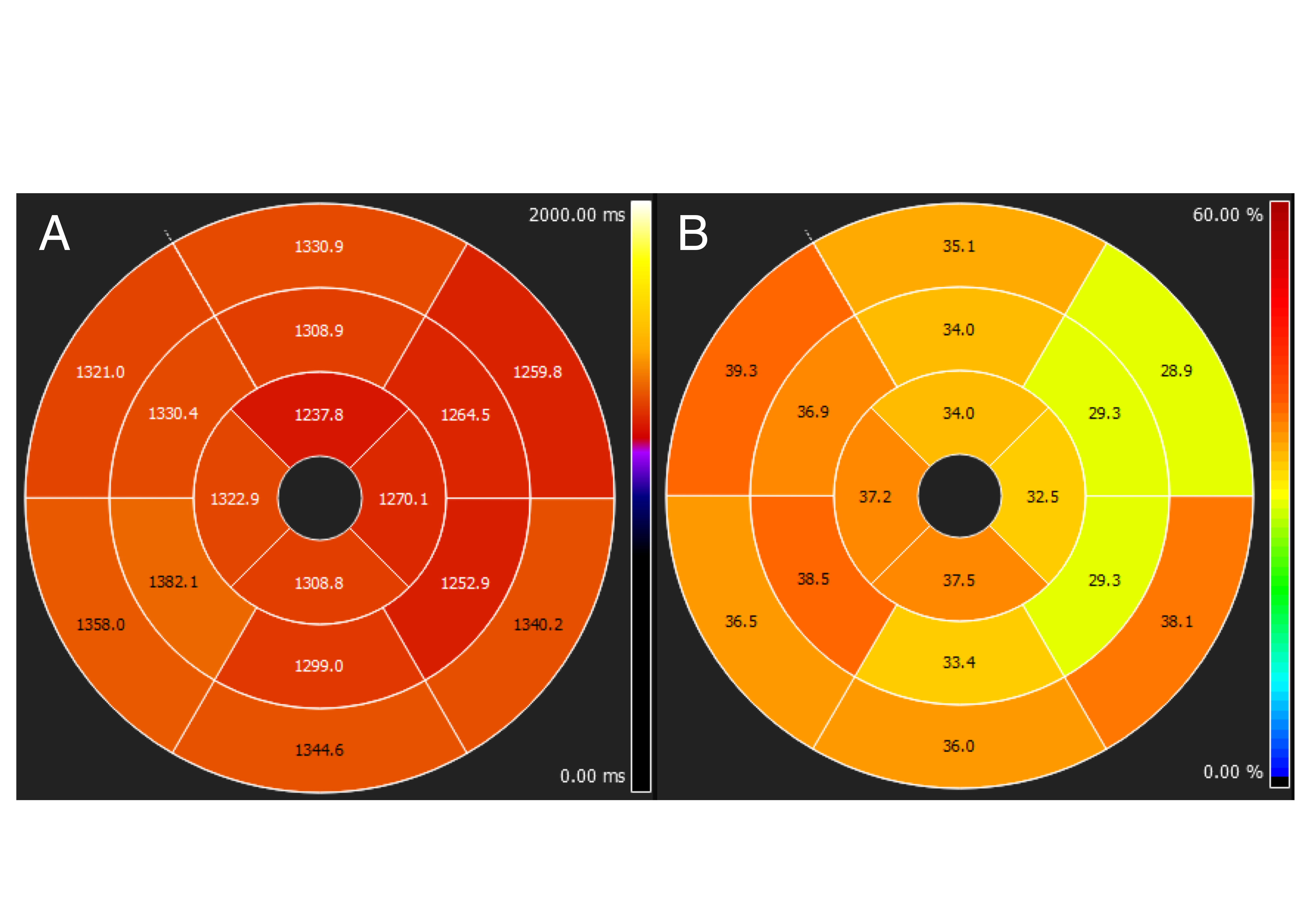

Supplement: ytaf029_Supplementary_Data [file ytaf029_supplementary_data.zip › Supplementary figure 2_R2.png]
